# The methodological and reporting characteristics of Campbell reviews: A systematic review

**DOI:** 10.1002/cl2.1134

**Published:** 2021-02-07

**Authors:** Xiaoqin Wang, Vivian Welch, Meixuan Li, Liang Yao, Julia Littell, Huijuan Li, Nan Yang, Jianjian Wang, Larissa Shamseer, Yaolong Chen, Kehu Yang, Jeremy M. Grimshaw

**Affiliations:** ^1^ Evidence‐based Medicine Centre of Lanzhou University Lanzhou China; ^2^ Clinical Epidemiology Program, Ottawa Hospital Research Institute Ottawa Ontario Canada; ^3^ Michael G. DeGroote Institute for Pain Research and Care, McMaster University Hamilton Ontario Canada; ^4^ Bruyère Research Institute Ottawa Ontario Canada; ^5^ Department of Health Research Methods, Evidence and Impact McMaster University Hamilton Ontario Canada; ^6^ Graduate School of Social Work and Social Research, Bryn Mawr College Bryn Mawr Pennsylvania USA; ^7^ School of Public Health, Lanzhou University Lanzhou China; ^8^ School of Public Health, University of Ottawa Ottawa Ontario Canada; ^9^ Li Ka Shing Knowledge Institute, St. Michael's Hospital Toronto Ontario Canada; ^10^ Department of Medicine University of Ottawa Ottawa Ontario Canada

## Abstract

**Background:**

The Campbell Collaboration undertakes systematic reviews of the effects of social and economic policies (interventions) to help policymakers, practitioners, and the public to make well‐informed decisions about policy interventions. In 2010, the Cochrane Collaboration and the Campbell Collaboration developed a voluntary co‐registration policy under the rationale to make full use of the shared interests and diverse expertise from different review groups within these two organizations. In order to promote the methodological quality and transparency of Campbell intervention reviews, the Methodological Expectations of Campbell Collaboration Intervention Reviews (MECCIR) were introduced in 2014 to guide Campbell reviewers. However, there has not been a comprehensive review of the methodological quality and reporting characteristics of Campbell reviews.

**Objectives:**

This review aimed to assess the methodological and reporting characteristics of Campbell intervention reviews and to compare the methodological quality and reporting completeness of Campbell reviews published before and after the implementation of MECCIR. A secondary aim was to compare the methodological quality and reporting completeness of reviews registered with Campbell only versus those co‐registered with Cochrane and Campbell.

**Search Methods:**

We searched the Campbell Library to identify all the completed intervention reviews published between 1 January 2011 to 31 January 2018.

**Selection Criteria:**

One researcher downloaded and screened all the records to exclude non‐intervention reviews based on reviews’ title and abstract. A second researcher checked the full text of all the excluded records to confirm the exclusion. In case of discrepancies, the two researchers jointly agreed on the final decision.

**Data Collection and Analysis:**

We developed the abstraction form based on mandatory reporting items for methods, results, and discussion from the MECCIR reporting standards Version 1.1; and additional epidemiological characteristics identified in a similar study of systematic reviews in health. Additionally, we judged the methodological quality and completeness of reporting of each included review. For methodological quality, we used the AMSTAR 2 (A MeaSurement Tool to Assess systematic Reviews 2) instrument; for reporting completeness we used the PRISMA (Preferred Reporting Items for Systematic Reviews and Meta‐Analyses) checklist. We rated reporting as either complete/partial or not reported. We described characteristics of the included reviews with frequencies and percentages, and median with interquartile ranges (IQRs). We used Stata version 12.0 to conduct multiple linear regressions for continuous data and the ordered logistic regressions for ordered data to investigate associations between prespecified factors and both methodological quality and completeness of reporting.

**Main Results:**

We included 96 Campbell reviews, 46 were published between January 2011 and September 2014 (pre‐MECCIR) and 50 between October 2014 and January 2018 (post‐MECCIR). Twenty‐two of 96 (23%) reviews were co‐registered with Cochrane. For overall methodological quality, 16 (17%) reviews were rated as high, 40 (42%) as moderate, 24 (25%) as low and 16 (17%) as critical low using AMSTAR 2. Reviews published after the release of MECCIR had better methodological quality ratings than those published before MECCIR (odds ratio [OR]   =6.61, 95% confidence interval [CI] [2.86, 15.27], *p* < .001). The percentages of reviews of high or moderate quality were 76% (post‐MECCIR) and 39% (pre‐MECCIR). Reviews co‐registered with Cochrane were rated as having better methodological quality than those registered only with Campbell (OR = 5.57, 95% CI [2.13, 14.58], *p* < .001). The percentages of reviews of high or moderate quality were 77% versus 53% between co‐registered and Campbell registered only reviews. Twenty‐five of 96 reviews (26%) completely or partially reported all 27 PRISMA checklist items. The median number of items reported across reviews was 25 (IQR, 22–26). Reviews published after the release of MECCIR reported 2.80 more items than those published before MECCIR (95% CI [1.74, 3.88], *p* < .001); reviews co‐registered on Campbell and Cochrane reported 1.98 more items than reviews only registered in Campbell (95% CI [0.72, 3.24], *p* = .003). An increasing trend over time was observed for both the percentage of high and moderate methodological quality of reviews and the median number of PRISMA items reported.

**Authors' Conclusions:**

Many features expected in systematic reviews were present in Campbell reviews most of the time. Methodological quality and reporting completeness were both significantly higher in reviews published after the introduction of MECCIR in 2014 compared with those published before. However, this may also reflect general improvement in the reporting the methodology of systematic reviews over time or associations with other characteristics which were not assessed such as funding or experience of teams. Reviews co‐registered with Cochrane were of higher methodological quality and more complete reporting than reviews only registered in Campbell.

## 1 PLAIN LANGUAGE SUMMARY

### 1.1 Methods and reporting of Campbell reviews are generally good: further improvement is possible

#### 1.1.1 The review in brief

Campbell systematic reviews of research on effectiveness of interventions are, in general, of high quality in terms of meeting international standards for the conduct and reporting of systematic reviews, though improvements could be made in several aspects.

Campbell reviews published after the introduction of the checklist of methodological expectation (Methodological Expectations of Campbell Collaboration Intervention Reviews [MECCIR]) in 2014 had better reporting and methodological qualities, however, this may be due to more global trends toward improvement over time.

#### 1.1.2 What is this review about?

The Campbell Collaboration undertakes systematic reviews of the effects of social and economic policies to help policymakers, practitioners, and the public to make well‐informed decisions about policy interventions. There has not been a comprehensive review of the methods and reporting characteristics of Campbell reviews. Nor has the methodological quality and completeness of reporting of Campbell reviews been assessed. These factors, which are assessed in this review, are important to ensure the transparency, reliability, and usability of Campbell systematic reviews.

#### 1.1.3 What is the aim of this review?

We collected information about the epidemiological, methodological, and reporting characteristics of Campbell reviews of the effects of policies published between 2001 and 2018, and assessed their methodological quality and completeness of reporting. We also assessed whether the methodological quality and completeness of reporting were better following the release of the MECCIR standards and in reviews that were co‐registered with Cochrane.

#### 1.1.4 What studies are included?

This review included 96 Campbell reviews that evaluated the effectiveness of any intervention. Forty‐six were published between January 2011 and September 2014 and 50 published between October 2014 and January 2018. Twenty‐two of the 96 reviews were co‐registered with Cochrane.

#### 1.1.5 What are the main findings of this review?

Fifty‐nine percent of the Campbell reviews were of high or moderate quality based on the AMSTAR 2. Twenty‐five of 96 (26%) Campbell reviews completely or partially reported all 27 PRISMA (Preferred Reporting Items for Systematic Reviews and Meta‐Analyses) checklist items. The median number of items reported across reviews was 25 (interquartile range [IQR], 22–26).

The methodological quality of Campbell reviews improved after the introduction of MECCIR, which might reflect a trend of improving quality over time. Seventy‐six percent of reviews published after the introduction of MECCIR had high or moderate methodological quality (AMSTAR 2) compared with 39% of reviews published before MECCIR.

Campbell reviews co‐registered with Cochrane had better methodological quality compared with those only registered on Campbell. Seventy‐seven percent of reviews registered in both Campbell and Cochrane were of high or moderate quality compared with 53% of reviews only registered with Campbell.

#### 1.1.6 What do the findings of this review mean?

Campbell reviews are generally well reported: 59% of the included reviews are of moderate to high methodological quality, according to PRISMA and AMSTAR 2 standards for systematic reviews. Campbell reviews published after MECCIR were of better quality than pre‐MECCIR, and reviews co‐registered in Cochrane scored better on quality indices than reviews only registered in Campbell.

This report may be used as a baseline for assessing effects of implementing strategies to address limitations identified by this review (such as interpreting risk of bias, reporting funding of individual studies, explaining the selection of study designs, citing the pre‐published protocol as well as other items). Future studies may be useful to explore more detail on specific methods such as search strategies and economic considerations.

#### 1.1.7 How up‐to‐date is this review?

This review included all the Campbell effectiveness reviews published between January 2011 and January 2018.

## 2 BACKGROUND

Systematic reviews aim to summarize “the best available research on a specific question by synthesizing the results of several studies” (Campbell Collaboration, 2018). They use transparent procedures to find, evaluate, and synthesize the results of relevant research whilst minimizing bias. They are increasingly popular across a wide range of sectors to inform policy and practice. Systematic reviews can support policymakers to develop evidence‐informed policy and help practitioners to keep up‐to‐date with relevant content knowledge (IOM, 2011; Oliver, 2015). In addition, granting agencies increasingly require the use of systematic reviews to justify new research. The trustworthiness of a systematic review is dependent upon the extent to which the review authors conducted the review using robust methods and the quality of reporting of the methods of the review (Steffen, 2010).

Moher et al. (2007) and Page et al. (2016) demonstrated poor conduct and highly variable reporting of systematic reviews in health‐related fields; they found only 7% of the included systematic reviews searched for unpublished data, less than half assessed the risk of publication bias and that the completeness of reporting was highly variable (Page et al., 2016). In the social sciences, Schalken and Rietbergen (2017) found reporting of quantitative systematic reviews in the field of industrial and organizational psychology was poor, for example, with only 1.7% reviews reporting assessment of individual study quality.

The Campbell Collaboration was established in 2001 to promote positive social and economic change through conducting and disseminating systematic reviews and other evidence syntheses. It undertakes systematic reviews to help policymakers, practitioners, and the public to make well‐informed decisions about policy interventions (V. Welch, 2018). The Campbell Collaboration has made several efforts to promote and improve its work. In 2010, Cochrane and the Campbell Collaboration developed a voluntary co‐registration policy under the rationale to make full use of the shared interests and diverse expertise from different review groups within these two organizations, avoid unnecessary duplication of effort by producing a single set of documents, and enable the availability to a wider audience (Campbell Systematic Reviews, 2010). This policy requires a well‐coordinated editorial process to produce reviews that meet more than one group's standards.

In order to promote the methodological quality and transparency of Campbell reviews of intervention effects, the MECCIR were introduced in 2014 (Chandler et al., 2017). Campbell review teams are strongly encouraged to conduct and report reviews following MECCIR. When submitting the protocol and completed review, reviewers are expected to provide a checklist affirming the adherence of MECCIR standards to the respective editor.

To date, we have little knowledge about the overall quality of methods and reporting of Campbell reviews. As such we conducted this study to investigate these aspects of Campbell reviews as well as to assess their methodological quality and completeness of reporting.

## 3 OBJECTIVES

The review has three main objectives:

1. To collect the methodological and reporting characteristics of Campbell reviews.
2. To assess the methodological quality and reporting completeness of Campbell reviews.
3. To determine the influence of MECCIR and co‐registration with Cochrane on the methodological quality and completeness of reporting of Campbell reviews.

## 4 METHODS

### 4.1 Title registration and protocol of the systematic review

The title registration and the protocol (Wang et al., 2019) for this systematic review were published in *Campbell Systematic Reviews* on 22 January 2019.

### 4.2 Criteria for considering studies for this review

We included completed Campbell intervention reviews published from January 2011 to January 2018. If a review had been updated, we selected the most recent version for inclusion. For reviews with additional objectives other than effectiveness (e.g., cost‐effectiveness), we only used data on intervention effectiveness. To explore how the MECCIR standards may have influenced the methodology and reporting of Campbell reviews, we compared all Campbell reviews published before the introduction of MECCIR (from January 2011 to September 2014) and all the Campbell reviews published after the introduction MECCIR (from October 2014 to January 2018). We did not consider a language restriction since all Campbell reviews are all published in English.

### 4.3 Search methods for identification of studies

We entered the *Campbell Systematic Reviews* portal through the official website of the Campbell Collaboration. We limited the search from 1 January 2011 to 31 January 2018 and downloaded the completed reviews from all review groups.

#### 4.3.1 Selection of studies

One researcher downloaded and screened all of the records to identify intervention effectiveness reviews (i.e., a review evaluating the effects of a social or policy intervention) and exclude non‐intervention reviews based on reviews’ title and abstract. A second researcher independently reviewed the full text of all the excluded records to confirm the exclusions. In the case of discrepancies, the two researchers arrived at the final decision through discussion.

#### 4.3.2 Data extraction and management

Four reviewers independently pilot tested the data extraction form. All reviewers abstracted two reviews in each pilot extraction and a third reviewer (X. W.) checked all the data for accuracy and consulted with a fourth reviewer (J. M. G.) where necessary. For the remaining extractions, two pairs of reviewers independently extracted data; discrepancies were resolved via discussion or adjudication by a third reviewer (X. W.).

We developed the abstraction form to collect information about methods and reporting of included reviews based on items from mandatory reporting items for methods and results from the MECCIR reporting standards Version 1.1 (MECCIR, 2017) and additional review characteristics used in a similar methodological study of reporting quality (Page et al., 2016).

We used Microsoft Excel 2018 to collect data on the following data extraction items:

1. epidemiological characteristics, including publication year, number and institute of authors, country of the first author, update status, co‐registration information, coordinating group, focus of the review, types of intervention, source of funding, and declaration of interest of authors;
2. reporting of title and abstract, including the term used in title and structured abstract;
3. reporting of the methods of reviews, including protocol preparation, data sources and search strategies, selection of studies, data collection, data analysis, and assessment of the risk of bias (ROB), etc.;
4. reporting of the results of reviews, especially results corresponding to the methods, including number of records retrieved and included, result of the analysis and assessment, etc.;
5. reporting of the discussion and other information, including summary of the main findings, relevance to key stakeholders, limitations at the study level and review level, implication for practice and future research, etc.

For points 2–5, we mainly noted whether the results were reported.

##### Quality assessment

###### Methodological quality

We used A MeaSurement Tool to Assess systematic Reviews 2 (AMSTAR 2) instrument (Shea et al., 2017) to evaluate the methodological quality of the included reviews. Four reviewers independently pilot tested the assessment tool. AMSTAR 2 contains 16 items (Box 1), including seven identified by the tool's authors as “critical” (meaning weaknesses in these items are critical and should reduce confidence in the findings of a review). For the assessment of the item related to protocol (i.e., item 2: establish the protocol in advance and justify the significant deviations from the protocol), we referred to the protocol of the review, if available. The individual items were categorized as yes, partial yes, no. For domains (i.e., domains 11, 12, and 15) concerning meta‐analysis, a “no meta‐analysis conducted” response was added for reviews not pooling data from individual studies (i.e. included no/insufficient studies, outcomes too variable to combine). A “not applicable” response was added for items 8–10 and 13–14 for reviews with no included studies (i.e., empty reviews) (Appendix A). The overall rating of confidence of each review was clarified as high, moderate, low, critically low considering the seven critical items (Shea et al., 2017) (Box 2). The detailed checklist and answers we uses are available in Appendix B. In addition, we referred to the AMSTAR 2 guidance document to help with the judgment on each item (https://www.bmj.com/content/bmj/suppl/2017/09/21/bmj.j4008.DC1/sheb036104.ww1.pdf).

Box 1. **AMSTAR 2 items**

Item 1: Did the research questions and inclusion criteria for the review include the components of PICO?

Item 2: Did the report of the review contain an explicit statement that the review methods were established prior to conduct of the review and did the report justify any significant deviations from the protocol?*

Item 3: Did the review authors explain their selection of the study designs for inclusion in the review?

Item 4: Did the review authors use a comprehensive literature search strategy?*

Item 5: Did the review authors perform study selection in duplicate?

Item 6: Did the review authors perform data extraction in duplicate?

Item 7: Did the review authors provide a list of excluded studies and justify the exclusions?*

Item 8: Did the review authors describe the included studies in adequate detail?

Item 9: Did the review authors use a satisfactory technique for assessing the risk of bias (RoB) in individual studies that were included in the review?*

Item 10: Did the review authors report on the sources of funding for the studies included in the review?

Item 11: If meta‐analysis was justified did the review authors use appropriate methods for statistical combination of results?*

Item 12: If meta‐analysis was performed did the review authors assess the potential impact of RoB in individual studies on the results of the meta‐analysis or other evidence synthesis?

Item 13: Did the review authors account for RoB in individual studies when interpreting/discussing the results of the review?*

Item 14: Did the review authors provide a satisfactory explanation for, and discussion of, any heterogeneity observed in the results of the review?

Item 16: Did the review authors report any potential sources of conflict of interest, including any funding they received for conducting the review?

Item 15: If they performed quantitative synthesis did the review authors carry out an adequate investigation of publication bias (small study bias) and discuss its likely impact on the results of the review?*

*Critical items

Box 2. **The overall rating of confidence of each review according to AMSTAR 2**

**High**

No more than one non‐critical weakness: the systematic review provides an accurate and comprehensive summary of the results of the available studies that address the question of interest

**Moderate**

More than one non‐critical weakness: the systematic review has more than one weakness but no critical flaws. It may provide an accurate summary of the results of the available studies that were included in the review

**Low**

One critical flaw with or without non‐critical weaknesses: the review has a critical flaw and may not provide an accurate and comprehensive summary of the available studies that address the question of interest

**Critically low**

More than one critical flaw with or without non‐critical weaknesses: the review has more than one critical flaw and should not be relied on to provide an accurate and comprehensive summary of the available studies

##### Completeness of reporting

We used the PRISMA checklist (Moher et al., 2009; Liberati et al., 2009) to evaluate the reporting quality of the included reviews. For each review, we judged each PRISMA item as “completely reported” or “not reported.” In addition, some PRISMA items had multiple components. For these items, we added an additional category of “partially reported” to indicate when only some of the components were reported. For example, a “partial yes” was given if a review only mentioned working from a protocol but did not say where to access the protocol, while a “yes” was given reported working from a protocol and provided the access (e.g., citation).

#### 4.3.3 Data synthesis

We summarized extracted data and quality assessments as frequencies and percentages for dichotomous data and median and IQR for continuous data. For methodological quality scores, we summarized the proportion of reviews of each rating category. For reporting completeness, we counted the number of PRISMA items reported in each review and report the median and IQR.

To explore whether AMSTAR 2 methodological quality ratings were influenced by MECCIR or co‐registration with Cochrane, we used ordered logistic regression to test the association between quality and the aforementioned factors, and reported associations using odds ratio and 95% confidence intervals. We excluded the “no meta‐analysis” and “not applicable” reviews when rating specific items.

To determine whether the number of PRISMA items reported in reviews was influenced by the introduction of MECCIR (pre‐ and post‐September 2014) and by co‐registration with Cochrane, we accumulated the overall number of PRISMA items reported: “1” if completely or not applicable, “0.5” if partially reported, and “0” if not reported. Then we used the multiple linear regression and report coefficients and 95% confidence interval.

All the analyses were conducted using Stata version 12.0.

#### 4.3.4 Differences between protocol and review

We planned to conduct subgroup analysis according to the introduction of MECCIR and co‐registration in our protocol. Instead, we used the logistic regressions to examine the potential interaction between these two factors.

## 5 RESULTS

### 5.1 Description of studies

#### 5.1.1 Results of the search

We ran the search in January 31, 2018 and identified 98 full Campbell reviews published since 2011. After reading all the full text, we included 96 reviews and excluded two non‐interventional reviews. The screening flow chart can be found in Appendix C.

#### 5.1.2 Included studies

Forty‐six reviews were published from January 2011 to September 2014 (pre‐MECCIR publication) and 50 published from October 2014 to February 2018 (post‐MECCIR publication). Most of the reviews had first authors from North America (41, 43%; mainly USA (37, 39%)) and Europe (49, 51%; mainly UK (26, 27%)). The 96 reviews were published by four coordinating groups within Campbell Collaboration: Social Welfare (37, 39%), International Development (28, 29%), Education (24, 25%), and Crime and Justice (22, 23%).

Reviews in our sample included a median of 18 (IQR, 7–38) studies. Ninety‐three reviews (97%) reviews considered experimental studies (i.e., randomized controlled trial [RCT], Quasi‐RCTs or other controlled experimental studies) and 13 (14%) considered observational studies as eligible study design (Table 1).

**Table 1:** Epidemiological characteristics of the included studies (96 reviews)

Characteristics	Category		Number (%)
Year of publication	2011		9 (9)
	2012		18 (19)
	2013		13 (14)
	2014		11 (11)
	2015		20 (21)
	2016		9 (9)
	2017		15 (16)
	2018		1 (1)
Country of corresponding author	North America	USA	37 (39)
		Canada	4 (4)
	Europe	UK	26 (27)
		Denmark	9 (9)
		Norway	7 (7)
		Netherlands	2 (2)
		Germany	2 (2)
		Belgium	1 (1)
		Ireland	1 (1)
		Switzerland	1 (1)
	Oceania	Australia	3 (3)
		New Zealand	1 (1)
	Africa	South Africa	2 (2)
Coordination group^a^	Social Welfare		37 (39)
	International Development		28 (29)
	Education		24 (25)
	Crime and Justice		22 (23)
	Nutrition		1 (1)
Files available on the Campbell website	Title form		69 (72)
	Protocol form		87 (91)
	Plain language summary		62 (65)
Registration	Co‐registered in Cochrane		22 (23)
	Campbell only		74 (77)
Update status	Original		80 (83)
	Updated		16 (17)
Number of included studies	0		5 (5)
	1–10		29 (30)
	11–20		17 (18)
	21–30		12 (13)
	31–40		10 (10)
	>40		23 (24)
Eligible study designs^b^	RCT		93 (97)
	Quasi‐RCTs		75 (78)
	Other controlled experimental studies^c^		45 (47)
	Observational studies (including cohort studies (4), case‐control studies (4), longitudinal observational non‐experimental studies (4), cross‐sectional studies (2), observational studies of unspecified type (2)		13 (14)
	Other (e.g. qualitative studies, quantitative evaluation studies, single subject design, studies with comparison)		5 (5)

^a^ 14 reviews belong to more than one group

^b^ 13 reviews included both experimental and observational studies

^c^ Other controlled experimental studies non‐RCTs, controlled before‐and‐after studies, interrupted time series studies.

Citations of the included reviews are available in supporting information Appendix D.

#### 5.1.3 Excluded studies

We excluded two non‐intervention reviews (see “Characteristics of excluded studies” section).

##### Methods and reporting characteristics of included reviews (Table 2)

**Table 2:** Methodological and reporting characteristics of included reviews (96)

		Publication date, *n* (%)		Registration, *n* (%)	
Reporting characteristics	All, *n* (%)	2011 Jan to 2014 Sep (46)	2014 Oct to 2018 Feb (50)	Campbell (74)	Campbell + Cochrane (22)
Protocol mentioned	85 (89)	37 (80)	48 (96)	63 (85)	22 (100)
Protocol cited in completed review	54 (56)	15 (32)	39 (78)	44 (59)	10 (45)
Protocol mentioned but not cited/no link in completed review	31 (32)	22 (48)	9 (18)	19 (26)	12 (55)
*Title and abstract*					
“Systematic review” or “meta‐analysis” used in title/abstract					
Systematic review (and meta‐analysis)	64 (67)	21 (46)	33 (86)	52 (70)	12 (55)
Not specified	32 (33)	25 (54)	7 (14)	22 (30)	10 (45)
Structured Abstract: All reviews reported the structured abstract					
*Methods*					
Study eligibility criteria: All reviews listed the eligibility criteria according to PICO factors					
Population	90 (94)	40 (87)	50 (100)	68 (92)	22 (100)
Intervention	96 (100)	46 (100)	50 (100)	74 (100)	22 (100)
Comparison	53 (55)	28 (61)	25 (50)	43 (58)	10 (45)
Outcome	94 (98)	44 (96)	50 (100)	72 (97)	22 (100)
Primary outcome specified	68 (71)	26 (57)	42 (84)	49 (66)	19 (86)
Harms considered	33 (34)	14 (30)	19 (38)	19 (26)	14 (63)
Setting/context	52 (54)	23 (50)	29 (58)	44 (59)	8 (36)
Eligible publication status		
Both published and unpublished studies	54 (56)	24 (52)	30 (60)	46 (62)	8 (36)
Not specified	42 (44)	22 (48)	20 (40)	28 (38)	14 (64)
Eligibility/ineligibility criteria based on study designs					
Eligible study designs specified	84 (88)	36 (78)	48 (96)	64 (86)	20 (91)
Eligible and ineligible study designs specified	10 (10)	8 (17)	2 (4)	8 (11)	2 (9)
Not specified	2 (2)	2 (4)	0 (0)	2 (3)	0 (0)
Study design					
Experimental (and quasi‐experimental) designs only	80 (83)	38 (83)	42 (84)	60 (81)	20 (91)
Experimental (and quasi‐experimental) + Observational designs	13 (14)	5 (11)	8 (16)	11 (15)	2 (9)
Not reported	3 (3)	3 (7)	0 (0)	3 (4)	0 (0)
Eligible languages					
No restrictions on languages	34 (35)	13 (28)	21 (42)	27 (36)	7 (31)
English only	10 (10)	9 (19)	1 (2)	8 (11)	2 (9)
English and other specific language(s)	7 (7)	1 (2)	6 (12)	7 (9)	0 (0)
Not specified	45 (47)	23 (50)	22 (44)	32 (43)	13 (59)
Information sources	96 (100)	46 (100)	50 (100)	74 (100)	22 (100)
Years of coverage reported on search					
Yes (start and end dates reported for all databases)	53 (55)	28 (61)	25 (50)	33 (45)	20 (91)
Partially (start and end dates reported for some databases, or only reported end date)	19 (20)	6 (13)	6 (13)	18 (24)	1 (5)
Not specified	24 (25)	12 (26)	12 (25)	23 (31)	1 (5)
Latest search date (within 12 months)					
Yes	24 (25)	12 (27)	12 (24)	14 (19)	10 (45)
No	70 (73)	32 (70)	38 (76)	58 (78)	12 (55)
Unclear	2 (2)	2 (4)	0 (0)	2 (3)	0 (0)
Search terms reported					
Full Boolean search logic reported for one or more database	85 (89)	40 (87)	45 (90)	63 (85)	22 (100)
Only free text words	11 (11)	6 (13)	5 (10)	11 (15)	0 (0)
Trial registry searched	40 (42)	23 (50)	17 (34)	28 (38)	12 (55)
Other sources	96 (100)	46 (100)	50 (100)	74 (100)	22 (100)
Gray literature	91 (95)	42 (91)	49 (98)	71 (96)	20 (91)
Reference lists	84 (88)	37 (80)	47 (94)	65 (88)	19 (86)
Hand searching particular journals	46 (48)	21 (46)	25 (50)	39 (53)	7 (32)
Conference abstracts	14 (16)	6 (13)	8 (16)	11 (15)	3 (14)
Contacting experts for additional studies	67 (70)	36 (78)	31 (62)	49 (66)	18 (82)
Screening, extraction, and risk of bias assessment methods					
Screening method	77 (80)	33 (72)	44 (88)	55 (74)	22 (100)
Data extraction method	89 (93)	43 (93)	46 (92)	67 (91)	22 (100)
Risk of bias (RoB)					
Assessment tool	79 (82)	31 (67)	48 (96)	57 (77)	22 (100)
Assessment method	60 (63)	25 (54)	35 (70)	40 (54)	20 (91)
Plan to contact corresponding authors	73 (76)	33 (72)	40 (80)	52 (70)	21 (95)
Statistical methods					
Plan for meta‐analysis	88 (92)	43 (93)	45 (90)	66 (89)	22(100)
Plan for statistical heterogeneity	77 (80)	35 (76)	42 (84)	56 (76)	21 (95)
Plan for risk of publication bias assessment	64 (67)	25 (54)	39 (78)	48 (65)	16 (73)
Additional analyses	81 (84)	39 (85)	42 (84)	59 (80)	22 (100)
Subgroup analysis	52 (54)	22 (48)	30 (60)	32 (43)	20 (91)
Sensitivity analysis	66 (69)	29 (63)	37 (74)	45 (61)	21 (95)
Meta‐regression	22 (23)	10 (22)	12 (24)	18 (24)	4 (18)
*Results*					
Review flow	87 (91)	40 (87)	47 (94)	69 (93)	18 (82)
Characteristics of included studies					
Table with details of each study	84 (88)	39 (85)	45 (90)	63 (85)	21 (95)
Summary information of all studies	7 (7)	4 (9)	3 (6)	7 (9)	0 (0)
Not applicable (no study included)	5 (5)	3 (7)	2 (4)	4 (5)	1 (5)
Total number of included participants					
Reported	19 (20)	12 (26)	7 (14)	10 (14)	9 (41)
Not reported	72 (75)	31 (67)	42 (82)	60 (81)	12 (55)
Not applicable (no study included)	5 (5)	3 (7)	2 (4)	4 (5)	1 (5)
Included studies listed					
Reported	89 (93)	43 (93)	46 (92)	68 (92)	21 (95)
Not reported	2 (2)	0 (0)	2 (4)	2 (3)	0 (0)
Not applicable	5 (5)	3 (7)	2 (4)	4 (5)	1 (5)
Excluded studies listed	89 (93)	42 (91)	47 (94)	67 (91)	22 (100)
Reasons for exclusion of full text					
Both in text and in flow diagram	78 (81)	38 (83)	40 (80)	60 (81)	18 (82)
Only in flow diagram	3 (3)	0 (0)	3 (6)	3 (4)	0 (0)
Only in text	12 (13)	5 (11)	7 (14)	8 (11)	4 (18)
Not reported	3 (3)	3 (7)	0 (0)	3 (4)	0 (0)
Result of RoB assessment					
Reported	76 (79)	30 (65)	46 (92)	55 (74)	21 (95)
Not reported	15 (16)	13 (28)	2 (4)	15 (20)	0 (0)
Not applicable	5 (5)	3 (7)	2 (4)	4 (5)	1 (5)
Result of publication bias assessment					
Reported	54 (56)	25 (54)	41 (82)	43 (58)	11 (50)
Not reported	19 (20)	12 (26)	7 (14)	14 (19)	5 (23)
Not applicable	23 (24)	3 (20)	2 (4)	17 (23)	6 (27)
Result of individual studies					
Reported	91 (95)	43 (93)	48(96)	70 (95)	21 (95)
Not applicable	5 (5)	3 (7)	2 (4)	4 (5)	1 (5)
Result of meta‐analysis					
Reported	81(84)	39 (85)	42 (84)	60 (81)	21 (95)
Not applicable	15 (16)	3 (7)	8 (16)	4 (19)	1 (5)
Results of additional analyses					
Reported	61 (64)	30 (65)	31 (62)	46 (62)	15 (68)
Not reported	9 (9)	5 (11)	4 (8)	9 (12)	0 (0)
Not applicable	26 (27)	11 (24)	15 (30)	19 (26)	7 (32)
*Discussion*					
Summarize the main findings	85 (89)	37 (80)	48 (96)	64 (86)	21 (95)
Relevance to key stakeholders	92 (96)	43 (93)	49 (98)	70 (95)	22 (100)
Account for RoB when interpreting results	70 (73)	28 (61)	42 (84)	50 (68)	20 (91)
Implication for future research	93 (97)	50 (100)	43 (93)	71 (96)	22 (100)
*Limitations, conclusions, COIs, and funding*					
Limitations reported	88 (91)	40 (87)	48 (96)	68 (92)	20 (91)
Limitations at both study and review level	59 (61)	20 (43)	39 (78)	46 (62)	13 (59)
Only limitations at the study level	19 (20)	13 (28)	6 (12)	16 (22)	3 (14)
Only limitations at the review level	10 (10)	7 (15)	3 (6)	6 (8)	4 (18)
COIs of SR authors	95 (99)	46 (100)	49 (98)	73 (99)	22 (100)
Source of funding of the SR	95 (99)	45 (98)	50 (100)	73 (99)	22 (100)
Role of funders for the SR	17 (18)	7 (15)	10 (20)	13 (18)	4 (18)

###### Protocol

Protocol was mentioned in 85 (89%) of included reviews, however, only 54 (56%) clearly cited the protocol in the completed review, 31 (32%) stated that they worked from a protocol but did not provide reference of the protocol (e.g., “XXX and XXX contributed to the development of this protocol”). Nine of the 11 reviews that did not mention a protocol had one available in the Campbell Library.

###### Title and abstract

Sixty‐four (67%) reviews were identified as a “systematic review” in the title, including four that described as a systematic review and meta‐analyses. All reviews had a structured abstract.

###### Methods

All included reviews reported the eligibility criteria according to the PICOS (population, intervention, comparison, outcome, setting) framework. All reviews included outcomes about the potential benefits of the intervention but only 33 (34%) reported potential harms. Most reviews (93 (97%)) specified the eligible study designs; 80 (83%) indicated that only experimental studies were eligible, and 13 (14%) specified that observational studies were also eligible. Fifty‐four (56%) reviews stated they would include published and unpublished studies. Seventeen (10%) reviews considered studies published in specific languages (English with or without other specific languages), and 34 (35%) indicated no limitations on language.

All reviews listed the information sources, while the details of the search strategy varied. For example, the years of search coverage were reported completely (i.e., both start and end dates were reported for all databases) in 53 (55%) of the reviews. Eighty‐five (89%) reviews reported the full Boolean search logic while some reviews only described free text words.

For the process of screening, extraction, and risk of bias assessment, 77 (80%), 89 (93%), and 60 (63%) reported the methods used, respectively. For statistical methods, 89 (93%) reviews reported their analysis plan and 77 (80%) described the methods for assessing statistical heterogeneity. Sixty‐four (67%) reviews reported their proposed methods to assess for publication bias and 81 (84%) reviews described planned approaches for additional analyses.

###### Results

Eighty‐seven (91%) reviews included a study flow diagram detailing the results of the screening process; 13 (14%) referred to the flow diagram as a PRISMA diagram. Ten reviews (10%) only summarized the results of the screening process in the review text and two (2%) did not report the results of the screening process. The included and excluded studies were listed in 89 (93%) reviews. Reasons for exclusion of full text articles were described in 92 (97%) reviews.

All reviews described the characteristics of included studies: 84 (88%) presented characteristics of individual studies, whereas seven (7%) only presented summary data across studies. For the 91 reviews which included one or more studies, only 19 (21%) reported the total number of included participants.

Seventy‐six (79%) reviews reported the result of risk of bias assessment, 47 (49%) used the Cochrane Risk of Bias tool, nine (9%) the Cochrane risk‐of‐bias tool for non‐randomized controlled studies, five (5%) the 3ie Risk of Bias tool, four IDCG risk of bias tool and four (4%) the Cochrane Effective Practice and Organisation of Care risk‐of‐bias tool. Thirty‐one (32%) reviews used other tools, or reported different aspects about risk of bias without using a specific tool. Publication bias assessment was reported in the results section of 54 (56%) reviews (including 10 reviews that did not describe their planned approach in the methods section). A further 20 (21%) reviews that described their planned approach to explore publication bias in the methods section were unable to do this because they included an insufficient number of studies.

###### Discussion and other information

The main findings and the strength of the evidence were summarized in the discussion section of 85 (89%) reviews. The relevance of the main findings to key stakeholders, such as policymakers, planners, practitioners (e.g., police, teachers, social workers) were mentioned in 92 (96%) reviews, and implications for future research were described in 93 (97%) reviews. Eighty‐eight (91%) reviews reported limitations, but only 59 (61%) described limitations at both the study and review level and limitations at the review level were mentioned less frequently than limitations of included studies.

##### Methodological quality assessment

**Figure 1:**
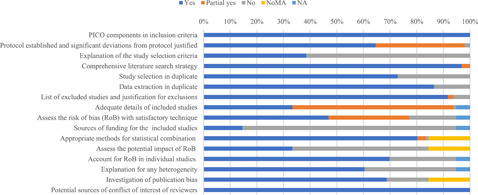
AMSTAR assessment of each item. NA, not applicable (for reviews which included no studies); NoMA, no meta‐analysis

Nine out of 16 AMSTAR 2 items were completely or partially addressed in more than 80% of the reviews (Figure 1). Less than 70% of reviews addressed five methodological items:

- Item 4: “Whether review authors explain their selection of the study designs for inclusion in the review?” (37, 39%).
- Item 10: “Whether review authors report on the sources of funding for the studies included in the review?” (14, 15%).
- Item 12: “If meta‐analysis was performed, whether review authors assess the potential impact of risk of bias in individual studies on the results of the meta‐analysis or other evidence synthesis?” (32, 33%).
- Item 14: “Did the review authors provide a satisfactory explanation for, and discussion of, any heterogeneity observed in the results of the review?” (58, 60%).
- Item 15: “If they performed quantitative synthesis, whether review authors carry out an adequate investigation of publication bias (small study bias) and discuss its likely impact on the results of the review?” (66, 69%).

For the methodological quality, 16 (17%) reviews were rated as high quality, 40 (42%) as moderate, 24 (25%) as low and 16 (17%) as critical low. The commonest AMSTAR 2 critical items not addressed were the consideration of ROB when interpreting the results of the review and assessing the presence and impact of publication bias. The percentages of high or moderate quality reviews were 76% versus 39% between post‐ and pre‐MECCIR (Table 3) and 77% versus 53% between Cochrane and Campbell co‐registered versus Campbell only registered.

**Table 3:** Overall confidence of AMSTAR 2 assessment (96) (overall, and according to pre‐/post‐MECCIR and whether co‐registered with Cochrane)

		Publication date		Registration	
Overall confidence	All	2011 Jan to 2014 Sep (46)	2014 Oct to 2018 Feb (50)	Campbell (74)	Campbell + Cochrane (22)
High	16 (17)	4 (9)	12 (24)	9 (12)	7 (32)
Moderate	40 (42)	14 (30)	26 (52)	30 (41)	10 (45)
Low	24 (25)	15 (33)	9 (18)	20 (27)	4 (18)
Critical low	16 (17)	13 (28)	3 (6)	15 (20)	1 (5)

Abbreviation: MECCIR, Methodological Expectations of Campbell Collaboration Intervention Reviews.

#### 5.1.4 Completeness of reporting assessment

**Figure 2:**
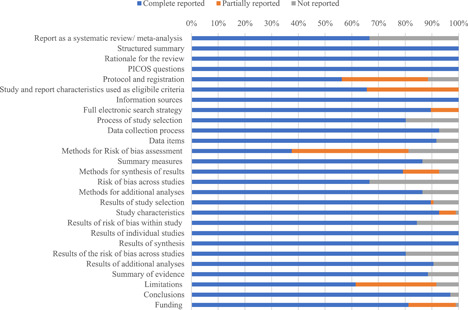
Percentage of PRISMA items reported in included reviews. PRISMA, Preferred Reporting Items for Systematic Reviews and Meta‐Analyses

Twenty‐five (26%) reviews reported or partially reported all 27 PRISMA checklist items. The median number of items reported was 25 (IQR, 22–26). Items about structured summary, rationale of review, PICOS questions, eligible criteria, information sources, search strategy, and results of synthesis were reported in all the reviews; while there are 33 (34%) of reviews only reported the eligible criteria regarding the study characteristics without mentioning the information on reports characteristics (e.g., publication languages, publication status, or study design), and 10 (10%) reviews only reported a summary of search terms without giving the full search strategy of any source of information. Items about title reported as a systematic review/meta‐analysis and methods of risk of bias across studies were reported in a relatively low percentage (less than 80%) of reviews compared with other PRISMA items (Figure 2).

#### 5.1.5 MECCIR and Cochrane co‐registration as predictors of the methodological quality and completeness of reporting of Campbell reviews

##### Methodological quality

After adjusting each factor using ordered logistic regression, reviews published after the release of MECCIR were of higher quality than those published before (odds ratio [OR] = 6.61, 95% confidence interval [CI] [2.86, 15.27], *p* < .001). Reviews co‐registered with Cochrane were of higher methodological quality than those only registered with Campbell (OR = 5.57, 95% CI [2.13, 14.58], *p* < .001) (Table 4).

**Table 4:** Ordered logistic regression of potential factors affecting methodological quality

		AMSTAR 2 assessment (n)					
Factors	Options (*n*)	CL	L	M	H	OR (95% CI)	*p*
Publication time	Post‐MECCIR (50)	3	9	26	12	6.61 (2.86, 15.27)	<.001
	Pre‐MECCIR (46)	13	15	14	4		
Registration	Cochrane + Campbell (22)	1	4	10	7	5.57 (2.13, 14.58)	<.001
	Campbell (74)	15	20	30	9		

Abbreviations: CI, confidence interval; CL, critical low; H, high; L, low; M, moderate; MECCIR, Methodological Expectations of Campbell Collaboration Intervention Reviews; OR, odds ratio.

We also graphed the percentage of all Campbell reviews that demonstrated in each quality level, by publication year (Figure 4). This showed overall improvement by time, especially after 2012.

**Figure 3:**
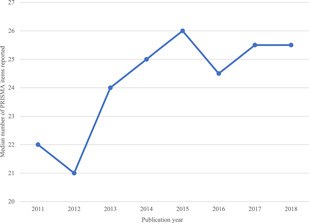
The median number of PRISMA items reported by year. PRISMA, Preferred Reporting Items for Systematic Reviews and Meta‐Analyses

**Figure 4:**
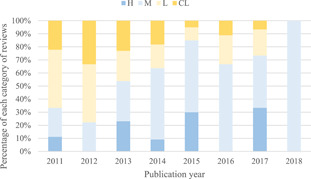
High and moderate quality reviews as a percentage of all Campbell reviews by publication year. CL, critical low; H, high; L, low; M, moderate

We also explored factors associated with ratings on the specific items of AMSTAR 2 using ordered logistic regression or ordinary logistic regression. When adjusting introduction of MECCIR or not, Campbell reviews that were co‐registered with Cochrane were more likely to be scored as meeting the full criteria for items about the protocol, selection in duplicate, risk of bias, sources of funding for the included studies, account for risk of bias and explanation for heterogeneity (Table 5a). When adjusting the co‐registration or not, reviews published after MECCIR were more likely to be scored as fully meeting the criteria for items about the protocol, rationale for selection of study design, risk of bias, and explanation for risk of bias (Table 5b). Fourteen and eight co‐registered reviews were published before MECCIR and after MECCIR, respectively.

**Table 5a:** Ordered logistic regression of potential factors affecting AMSTAR 2 items

	Cochrane + Campbell (*n* (%))			Campbell (*n* (%))			Cochrane + Campbell vs. Campbell		
Item	Y	PY	N	Y	PY	N	OR	*p*	95% CI
1. PICO components	All provided research questions and inclusion criteria with PICO components								
2. Protocol^a^	18 (82)	4 (18)	0	44 (59)	28(38)	2 (3)	5.52	.009*	(1.53, 19.95)
3. Explain for selection of study design	6 (27)	0	16 (73)	31 (42)	0	43 (58)	0.66	.457	(0.22, 1.99)
4. Search strategy^a^	22 (100)	0	0	71 (96)	3(4)	0	1.39E+07	.997	(0.00, NE)
5. Selection in duplicate	20 (91)	0	2 (9)	50 (68)	0	24 (32)	6.27	.023*	(1.28, 29.73)
6. Data extraction in duplicate	21 (95)	0	1 (5)	62 (84)	0	12 (16)	4.46	.167	(0.54, 37.03)
7. List of excluded studies^a^	22 (100)	0	0	66 (89)	2(3)	6 (8)	1.69E+07	.995	(0.00, NE)
8. Details of included studies	10 (48)	11 (52)	0	22 (31)	47(67)	1 (1)	2.02	.169	(0.74, 5.51)
9. Risk of bias^a^	16 (76)	5 (24)	0	29 (41)	24(34)	17 (24)	8.34	.001*	(2.51, 27.79)
10. Sources of funding for the included studies	7 (33)	0	14 (67)	7 (10)	0	63 (90)	5.65	.008*	(1.58, 20.22)
11. Methods for statistical analysis^a^	21 (100)	0	0	56 (93)	3(5)	1 (2)	5,052,027	.994	(0.00, NE)
12. Impact of RoB	12 (57)	0	9 (43)	20 (33)	0	40 (67)	3.37	.028*	(1.13, 10.01)
13. Account for RoB^a^	20 (95)	0	1 (5)	47 (67)	0	23 (33)	21.79	.005*	(2.53, 187.88)
14. Explanation for heterogeneity	18 (86)	0	3 (14)	40(57)	0	30 (43)	5.66	.012*	(1.46, 21.95)
15. Publication bias^a^	16 (76)	0	5 (24)	50 (83)	0	10 (17)	0.74	.141	(0.21, 2.58)
16. Sources of COI	All reported potential sources of conflict of interest								

Abbreviations: CI, confidence interval; N, no; NE, not estimable; OR, odds ratio; PY, partial yes; Y, yes.

^a^ Critical items.

* *p* < .05.

**Table 5b:** Ordered logistic regression of potential factors affecting AMSTAR 2 items

	After 2014 Sep (*n* (%))			After 2014 Sep (*n* (%))			After vs. Before 2014 Sep		
Item	Y	PY	N	Y	PY	N	OR	*p*	95% CI
1. PICO components	All provided research questions and inclusion criteria with PICO components								
2. Protocol^a^	41 (82)	8 (16)	1 (2)	21 (46)	24 (52)	1 (2)	7.22	<.001*	(2.68, 19.50)
3. Explain for selection of study design	27 (54)	0	23 (46)	10 (22)	0	36 (78)	4.02	.003*	(1.63, 9.92)
4. Search strategy^a^	48 (96)	2 (4)	0	45 (98)	1 (2)	0	0.65	.725	(0.05, 7.45)
5. Selection in duplicate	40 (80)	0	10 (20)	30 (65)	0	16 (35)	2.74	.041*	(1.04, 7.20)
6. Data extraction in duplicate	44 (88)	0	6 (12)	39 (85)	0	7 (15)	1.54	.476	(0.47, 5.11)
7. List of excluded studies^a^	45 (90)	2 (4)	3 (6)	43 (93)	0	3 (7)	0.8	.775	(0.18, 3.63)
8. Details of included studies	16 (33)	32 (67)	0	16 (37)	26 (60)	1 (2)	1	.996	(0.42, 2.41)
9. Risk of bias^a^	17 (40)	12 (28)	14 (33)	28 (58)	17 (35)	3 (6)	4.41	.001*	(1.80, 10.78)
10. Sources of funding for the included studies	9 (19)	0	39 (81)	5 (12)	0	38 (88)	2.54	.159	(0.69, 9.26)
11. Methods for statistical analysis^a^	39 (93)	3 (7)	0	38 (97)	0	1 (3)	0.44	.485	(0.04, 4.46)
12. Impact of RoB	20 (48)	0	22 (52)	12 (31)	0	27 (69)	2.6	.057	(0.97, 6.93)
13. Account for RoB^a^	44 (92)	0	4 (8)	23 (53)	0	20 (47)	15.81	<.001*	(4.46, 56.08)
14. Explanation for heterogeneity	34 (71)	0	14 (29)	24 (56)	0	19 (44)	2.49	.053	(0.99, 6.2)
15. Publication bias^a^	37 (88)	0	5 (12)	29 (74)	0	10 (26)	2.44	.141	(0.74, 8.06)
16. Sources of COI	All reported potential sources of conflict of interest								

Abbreviations: CI, confidence interval; N, no; NE, not estimable; OR, odds ratio; PY, partial yes; Y, yes.

^a^ Critical items.

* *p* < .05.

##### Completeness of reporting

Based on multiple linear regression analysis, reviews published after the release of MECCIR reported 2.80 items more than those published before MECCIR (95% CI [1.74, 3.88], *p* < .001). Reviews co‐registered with Campbell and Cochrane reported 1.98 more PRISMA items than reviews only registered with Campbell (95% CI [0.72, 3.24], *p* = .002) (Table 6).

**Table 6:** Multiple linear regression analysis of potential factors affecting reporting of PRISMA items

		Number of PRISMA items reported			
Factors	Options	Mean (*SD*)	Median (interquartile ranges)	Number of additional items reported (95% CI)	*p*
Publication time	Post‐MECCIR (50)	24.53 (0.31)	25.50 (24.00–26.00)	2.80 (1.74, 3.88)	<.001
	Pre‐MECCIR (46)	22.01 (0.46)	23.00 (20.50–24.38)		
Registration	Cochrane + Campbell (22)	24.41 (0.34)	24.50 (24.00–25.50)	1.98 (0.72, 3.24)	.002
	Campbell (74)	23.00 (0.37)	24.00 (21.13–25.50)		

Abbreviations: CI, confidence interval; MECCIR, Methodological Expectations of Campbell Collaboration Intervention Reviews; PRISMA, Preferred Reporting Items for Systematic Reviews and Meta‐Analyses.

We also graphed the median number of PRISMA items reported by publication year (Figure 3), which showed a steady improvement in reporting completeness from 2012 to 2015.

## 6 DISCUSSION

### 6.1 Summary of main results

We evaluated the methodological and reporting characteristics of 96 Campbell reviews: forty‐six were published before MECCIR and 50 published after MECCIR standards were introduced. Twenty‐two reviews were co‐registered with Cochrane. Over 90% of these reviews were carried out by researchers from high‐income countries; the remaining two reviews were from South Africa (upper‐middle income country).

When considering reporting, a median of 25 out of the 27 PRISMA items were reported. Since all of PRISMA items are included in MECCIR, except for the title, it is not surprising that these items were well‐reported. For specific characteristics, 33% of reviews did not report the title as a systematic review, this might be because all the Campbell reviews were in *Campbell Systematic Reviews* and Campbell does not require this. For some sections, the reported details varied, such as the details of search strategy and the characteristics of the included studies. Forty‐nine percent of completed reviews failed to cite the protocol. Nearly half of the reviews failed to report on whether unpublished studies were included in the review, despite its relevance for systematic reviews (Trespidi et al., 2011).

According to AMSTAR 2, 59% of the Campbell reviews were rated as high or moderate quality. We identified several areas for improvement in methods including clarification of the rationale for including specific study design, technique for assessing the risk of bias, the sources of funding for the included studies, and assessment of the potential impact of ROB in individual studies on the evidence synthesis. Whilst MECCIR recommends risk of bias assessment for RCT using the Cochrane risk of bias tool (Higgins et al., 2011), it provides no guidance for assessing ROB in non‐RCT (MECCIR 2017). Since many Campbell reviews included study designs other than RCTs, Campbell review authors may need to pay more the attention to assessing ROB‐related standards for different study designs. AMSTAR 2.0 includes two new items (funding sources of included studies and impact of ROB in individual studies); these were only achieved in 15% and 24% of the reviews, a relatively low proportion compared with the other items. This might imply that these methodological issues did not receive enough attention before (Shea et al., 2017).

Both methodological quality and completeness of reporting were better after the introduction of MECCIR, possibly because MECCIR clarified expectations for authors and editors and provided structured tools for assessing the reporting and methodology of reviews which could be used by both editors and authors. These improvements may also reflect general improvements in the reporting of systematic reviews over time due to external factors such as increasing awareness of the importance of transparency and reproducibility.

Our findings also showed that reviews co‐registered with Cochrane were of better methodological quality and had more complete reporting than those only registered with Campbell. This is possibly because of the impact of the coordinated editorial and peer review processes taking place under the collaboration of Campbell and Cochrane.

### 6.2 Overall completeness and applicability of evidence

This methodological review includes all Campbell intervention reviews published from January 2011 to January 2018. The evidence provided here will help future Campbell reviews (and potentially non‐Campbell Reviews) address known deficiencies in methodological quality and completeness of reporting of policy reviews. As we did not include non‐intervention reviews, we do not know whether they have similar characteristics and cannot comment on the applicability of these findings to non‐intervention reviews.

We also did not aim to compare Campbell reviews with non‐Campbell reviews in this study. As such we are unable to comment on the similarity and differences in methodological and reporting characteristics of non‐Campbell reviews in the social sciences.

### 6.3 Potential biases in the review process

To ensure the reliability of the data extraction and quality assessment, we appraised the studies in duplicate, and a third reviewer was consulted if there were discrepancies. The observed improvement in methodological quality and completeness of reporting following the introduction of MECCIR may be confounded by general improvements in these over time Also, there may be other characteristics driving the effects seen post‐MECCIR. For example, the author teams might be more experienced, the reviews may have had more funding and therefore had more resources to prepare their reports, and more Campbell resources may have been available. Future studies are needed to analyze the potential impact of these factors. Also, for the completeness of reporting assessments, we considered partially reported items as 0.5, while some items may have more than two components and the importance of these components may be different to each other. Therefore our estimates of reporting completeness may not reflect the potential range of reporting completeness across Campbell reviews. Other studies have similar problems (Page & Moher, 2017).

### 6.4 Agreements and disagreements with other studies or reviews

Researchers from China analyzed the publication characteristics of Campbell reviews in 2017. The study only described some basic characteristics including where the reviewers came from and what kind of software was used for data management and provided limited insight on other methodological characteristics (Shang et al., 2019). Schalken and Rietbergen (2017) analyzed systematic reviews on industrial and organizational psychology, where the Meta‐Analysis Reporting Standards (MARS) were used to assess reporting in the method section only. The results indicated unsatisfactory overall reporting of included reviews: the eligible population and research design were reported in 29% and the search time period were fully reported in 23% of reviews (Schalken & Rietbergen, 2017).

The quality of some AMSTAR items was low including explaining the rationale for the study designs for inclusion, selecting the individual studies in duplicate, stating the sources of funding for included studies, assessing potential impact of risk of bias of individual studies on meta‐analyses, and investigating publication bias. Similar findings were also observed among other systematic reviews (Almeida et al., 2019; Anaya et al., 2019; Cortese et al., 2019; Pussegoda et al., 2017; Yan et al., 2019).

When compared to 45 Cochrane reviews evaluated in a similar methodological study by Page et al. (2016), both Cochrane and Campbell reviews had similar levels of reporting information about registration and protocol (98% vs. 89%), conflict of interest of review authors (100% vs. 99%), and source of funding for the review(96% vs. 99%) and assessing statistical heterogeneity and publication bias (both around 80%). However, Cochrane reviews did better than Campbell reviews on several aspects including reporting the eligible publication status (96% vs. 56%) and eligible language (87% vs. 53%) of included studies, screening methods (100% vs. 80%), and total number of participants (93% vs. 21%). When compared with all the overall PRISMA assessment across 27 studies (mostly focused on therapeutic interventions and/or diagnosis) published from 2010 to 2016 that have assessed 2382 SRs (Page & Moher, 2017), Campbell reviews had more complete reporting on many of the aspects, especially the structured summary (100% vs. 79%), protocol and registration (56% vs. 21%), information sources (100% vs. 84%), additional analysis (86% vs. 60%), and funding sources for systematic reviews (81% vs. 60%). Campbell reviews also appeared to be less completely reported than other reviews in some areas, including identifying the report as a systematic review (67% vs. 87%) and the limitations (61% vs. 80%). In addition, methods for risk of bias across studies was reported in a relatively low proportion of systematic reviews for both Campbell and non‐Campbell groups (67% vs. 59%) (Page & Moher, 2017).

## 7 AUTHORS' CONCLUSIONS

Our study provides comprehensive and valuable information about systematic reviews across the social sciences. First, we included Campbell reviews without restriction on the research fields, reviews on Social Welfare, International Development, Education and Crime and Justice were all included. Second, we examined both the methodological and reporting quality, which is informative and valuable for researchers and for the Campbell Collaboration to make improvements in the future work, toward providing more transparent and reliable evidence to policymakers, practitioners, and the public in the future.

The methodological quality of Campbell reviews was good when compared to other reviews (Almeida et al., 2019; Anaya et al., 2019; Cortese et al., 2019; Page & Moher, 2017; Pussegoda et al., 2017; Yan et al., 2019). AMSTAR 2 ratings show that 59% of Campbell reviews were of high or moderate quality, and the number of reviews of low and critical low quality was decreasing by time. Methodological details were often lacking about the rationale for including specific study designs, using appropriate techniques to assess the risk of bias, presenting the funding sources for the included studies, and assessing the potential impact of ROB on the evidence synthesis. Campbell reviews in our sample were well reported, with a median of 25 out of 27 (93%) of PRISMA items reported. Inadequate details were observed in specific aspects, including citing the protocol, clarifying the search dates (start and end dates, latest search date), listing and justifying the excluded studies, and describing the included studies adequately. Methodological quality and reporting completeness were both significantly higher in reviews published after the introduction of MECCIR in 2014 compared to those published before; and reviews co‐registered with Cochrane was of higher methodological quality and better completeness of reporting than reviews only registered in Campbell. However, the improvement might also be related with some other factors, such as time.

### 7.1 Implications for practice

Our study could help Campbell reviewers, peer reviewers, and editors be aware of areas for improvement, in particular reporting the search dates, citing the protocol and reporting the deviations from protocol, and providing the characteristics of the included studies. These findings may also be useful for refining the Campbell Revman templates to guide reporting. Meanwhile, the Campbell editors may consider developing more guidance in particular areas including the ROB assessment of the included studies, and justification of excluded studies. In addition, the Campbell reviewers as well as Campbell coordinating groups need to pay more attention to assessment and interpretation of risk of bias, rationale for selection of study design, investigation of publication bias, as well as the new methodological AMSTAR 2 items about the funding sources for the included studies and the potential impact of ROB in individual studies on the synthesis results.

Campbell has a structured process for reviewers to follow, from title to protocol registration, and then submit the full review. Reviewers should plan for the protocol rigorously and clearly so as to ease the conduct of the full review, and report the final result transparently and completely to make it more accessible. Editors might consider making the MECCIR reporting standards more user‐friendly to make it easy to follow (e.g., integrate the important standards in the Review Template). Open synthesis (Haddaway, 2018) has been encouraged to improve research transparency and Campbell also encourages open synthesis (V. A. Welch, 2019), which could benefit for future studies.

### 7.2 Implications for research

This review provides an initial baseline assessment of overall reporting characteristics and methodological quality of Campbell reviews. Future studies may be useful to appraise specific processes in more detail such as search strategy development and how to consider economic evaluation.

The Campbell Collaboration appointed its first full‐time editor in chief in late 2017 which may have an impact on reporting completeness and methodological quality of coming Campbell reviews by focusing on editorial consistency and quality standards across groups. Future studies could examine whether the methodological quality and completeness of reporting continue to improve over time.

The PRISMA working group has recently updated the PRISMA 2009 as PRISMA 2020 (Page et al., 2020). It provides detailed essential elements and additional elements for each item, which is clearer for reviewers to use. Future studies could have deeper exploration based on the detailed elements. In addition, studies to explore the effectiveness of interventions to improve the adherence of MECCIR standards and to improve the reporting and methodological quality could also be helpful. Campbell already requires authors submit MECCIR checklists and expects articles to be MECCIR compliant. Endorsement and implementation of reporting guidelines in the editorial process such as this has been shown to improve adherence to reporting guidelines (Turner et al., 2012). This study identifies priority items which need additional verification in the editorial process with the MECCIR checklist such as citing the protocol, clearly describing the methods and results of risk of bias assessment, reporting the role of the funding source of reviews.

## AUTHOR CONTRIBUTIONS

- Content: Xiaoqin Wang, Jeremy Grimshaw, Vivian Welch
- Systematic review methods: Vivian Welch, Jeremy Grimshaw, Julia Littell, Kehu Yang, Larissa Shamseer, Yaolong Chen
- Statistical analysis: Xiaoqin Wang, Vivian Welch, Liang Yao
- Information retrieval: Xiaoqin Wang, Nan Yang, Huijuan Li, Meixuan Li, Jianjian Wang

## CONFLICT OF INTERESTS

Vivian Welch is the editor in chief of the Campbell Collaboration, Jeremy Grimshaw is the President of the board of the Campbell Collaboration, Julia Littell is a member of the Technical Advisory Group of the Campbell Collaboration.

## PUBLISHED NOTES

### Characteristics of studies

#### Characteristics of included studies

See supporting information Appendix D for the Excel for the Characteristics of included studies.

**Characteristics of excluded studies**

**Table:** 

Title	ID	Coordinating group	Reason for exclusion
Preschool predictors of later reading comprehension ability	Hjetland H, 2017	Education group	Not effectiveness review
The relationship between teacher qualification and the quality of the early childhood care and learning environment	Manning M, 2017	Education group	Not effectiveness review

## SOURCES OF SUPPORT

**Internal sources**

- No sources of support provided

**External sources**

Xiaoqin Wang is supported by the China Scholarship Council.

Vivian Welch is supported by an Ontario Early Researcher Award (2014–2019).

Julia Littell is supported by Bryn Mawr College.

Jeremy Grimshaw is supported by a Canada Research Chair in Health Knowledge Transfer and Uptake.

Kehu Yang is supported by the Major Project of the National Social Science Fund of China: "Research on the Theoretical System, International Experience and Chinese Path of Evidence‐based Social Science" (Project No. 19ZDA142).

## Supporting information

Supporting information

## APPENDIX A REVIEWS INCLUDED NO STUDIES OR WITHOUT META‐ANALYSIS

**Table:**

1. Parker, B., & William, T. Psychoanalytic/psychodynamic psychotherapy for children and adolescents who have been sexually abused. *Campbell Systematic Reviews*. 2013, *9*(1), i–58. https://doi.org/10.4073/csr.2013.13	Blank review
2. Westbrook, J., Fong, C., Nye, C, Williams, A, Wendt, O, & Cortopassi, T. Pre‐graduation transition services for persons with autism spectrum disorders: effects on employment outcomes. *Campbell Systematic Reviews*. 2013, *9*(1), 1–70. https://doi.org/10.4073/csr.2013.11	Blank review
3. van der Laan, P., Smit, M., Busschers, I., & Aarten, P. Cross‐border trafficking in human beings: Prevention and intervention strategies for reducing sexual exploitation. *Campbell Systematic Reviews*. 2011, *7*(1), 1–50. https://doi.org/10.4073/csr.2011.9	Blank review
4. Ott, E., & Montgomery, P. Interventions to improve the economic self‐sufficiency and well‐being of resettled refugees: A systematic review. *Campbell Systematic Reviews*. 2015, *11*(1), 1–53. https://doi.org/10.4073/csr.2015.4	Blank review
5. Higginson, A., Benier, K., Shenderovich, Y., Bedford, L., Mazerolle, L., & Murray, J. Preventive interventions to reduce youth gang violence in low‐ and middle‐income countries: A systematic review. *Campbell Systematic Reviews*. 2015, *11*(1), 1–176. https://doi.org/10.4073/csr.2015.18	Objective 1 (intervention effectiveness) and Objective 2 (reasons for implementation success or failure); no studies included on objective 1
6. Doocy, S., & Tappis, H. Cash‐based approaches in humanitarian emergencies: A systematic review. *Campbell Systematic Reviews*, 2017, *13*(1). https://doi.org/10.4073/csr.2017.17	Included studies without conducting meta‐analysis
7. Vigurs, Carol. Interventions to improve the labour market situation of adults with physical and/or sensory disabilties in low‐ and middle‐income countries: A systematic review. *Campbell Systematic Reviews*. 2015, 20. *Campbell Collaboration*, 2015,11(1). https://doi.org/10.4073/csr.2015.20	Included studies without conducting meta‐analysis
8. Trine Filges, Ditte Andersen, Anne‐Marie, Klint Jørgensen. Functional family therapy (FFT) for young people in treatment for non‐opioid drug use. *Campbell Collaboration*. 2015,11(1). https://doi.org/10.4073/csr.2015.14	Included studies without conducting meta‐analysis
9. Sabine Wollscheid, Heather Menzies, Munthe‐Kaas, Karianne Thune Hammerstrøm, & Eamonn Noonan. Effect of interventions to facilitate communication between families or single young people with minority language background and public services. *Campbell Collaboration*. 2015,*11*(1). https://doi.org/10.4073/csr.2015.7	Included studies without conducting meta‐analysis
10. Samii, C., Lisiecki, M., Kulkarni, P., et al. Effects of decentralized forest management (DFM) on deforestation and poverty in low‐ and middle‐ income countries: A systematic review. *Campbell Collaboration*. 2014, *10*(1). https://doi.org/10.4073/csr.2014.10	Included studies without conducting meta‐analysis
11. Tolan, P., Henry, D., Schoeny, M., et al. Mentoring interventions to affect juvenile delinquency and associated problems. *Campbell Collaboration*. 2013, *9*(1). https://doi.org/10.4073/csr.2013.10	Included studies without conducting meta‐analysis
12. Ulrik Gensby, Thomas Lund, Krystyna Kowalski, et al. PROTOCOL: Workplace disability management programs promoting return‐to‐work (RTW). *Campbell Systematic Reviews*. 2011, *7*(1). https://doi.org/10.1002/CL2.83	Included studies without conducting meta‐analysis
13. Westbrook, J. D., Martin, F. H., Nye, C., et al. PROTOCOL: Effectiveness of adult employment assistance services for persons with autism spectrum disorders. *Campbell Systematic Reviews*. 2012, *6*(1). https://campbellcollaboration.org/better-evidence/employment-assistance-services-persons-with-autism.html	Included studies without conducting meta‐analysis
14. Christopher S. Koper. PROTOCOL: Police strategies for reducing illegal possession and carrying of firearms. *Campbell Systematic Reviews*. 2012. https://doi.org/10.4073/csr.2012.11	Included studies without conducting meta‐analysis
15. David B. Wilson, David Weisburd, & David McClure. Use of DNA testing in police investigative work for increasing offender identification, arrest, conviction and case clearance. *Campbell Systematic Reviews*. 2011 (7). https://doi.org/10.4073/csr.2011.7	Included studies without conducting meta‐analysis

## APPENDIX B AMSTAR 2 ASSESSMENT TOOL

**AMSTAR 2 appraisal tool and examples**

**Table:**

**1. Did the research questions and inclusion criteria for the review include the components of PICO?**		
For Yes:	Optional (recommended)	
▯ Population	▯ Timeframe for follow‐up	▯ Yes
▯ Intervention		
▯ Comparator group		▯ No
▯ Outcome		
**2. Did the report of the review contain an explicit statement that the review methods were established prior to the conduct of the review and did the report justify any significant deviations from the protocol?** *		
For Partial Yes:	For Yes:	▯ Yes
The authors state that they had a written protocol or guide that included ALL the following:	As for partial yes, plus the protocol should be registered and should also have specified:	
▯ review question(s)	▯ a meta‐analysis/synthesis plan, if appropriate, *and*	▯ Partial Yes
▯ a search strategy	▯ a plan for investigating causes of heterogeneity	▯ No
▯ inclusion/exclusion criteria	▯ justification for any deviations from the protocol	
▯ a risk of bias assessment	
**3. Did the review authors explain their selection of the study designs for inclusion in the review?**		
For Yes, the review should satisfy ONE of the following:		
▯ *Explanation for* including only RCTs		▯ Yes
▯ OR *Explanation for* including only NRSI		▯ No
▯ OR *Explanation for* including both RCTs and NRSI		
**4. Did the review authors use a comprehensive literature search strategy?** *		
For Partial Yes (all the following):	For Yes, should also have (all the following):	
	searched the reference lists/bibliographies of included studies	▯ Yes
▯ searched at least 2 databases (relevant to research question)	▯ searched trial/study registries	▯ Partial Yes
	▯ included/consulted content experts in the field	▯ No
▯ provided key word and/or search strategy	▯ where relevant, searched for grey literature	
▯ justified publication restrictions (e.g. language)	▯ conducted search within 24 months of completion of the review
**5. Did the review authors perform study selection in duplicate?**		
For Yes, either ONE of the following:		▯ Yes
▯ at least two reviewers independently agreed on selection of eligible studies and achieved consensus on which studies to include		▯ No
▯ OR two reviewers selected a sample of eligible studies and achieved good agreement (at least 80 percent), with the remainder selected by one reviewer.		
**6. Did the review authors perform data extraction in duplicate?**		
For Yes, either ONE of the following:		
▯ at least two reviewers achieved consensus on which data to extract from included studies		▯ Yes
▯ OR two reviewers extracted data from a sample of eligible studies and achieved good agreement (at least 80 percent), with the remainder extracted by one reviewer.		▯ No
**7. Did the review authors provide a list of excluded studies and justify the exclusions?** *		
For Partial Yes:	For Yes, must also have:	▯ Yes
▯ provided a list of all potentially relevant studies that were read in full‐text form but excluded from the review	▯ Justified the exclusion from the review of each potentially relevant study	▯ Partial Yes
		▯ No
**8. Did the review authors describe the included studies in adequate detail?**		
For Partial Yes (ALL the following):	For Yes, should also have ALL the following:	▯ Yes
		▯ Partial Yes
▯ described populations	▯ described population in detail	▯ No
▯ described interventions	▯ described intervention in detail (including doses where relevant)	
▯ described comparators	▯ described comparator in detail (including doses where relevant)	▯ Not applicable (no study included) #
▯ described outcomes	▯ described study's setting	
▯ described research designs	▯ timeframe for follow‐up	
**9. Did the review authors use a satisfactory technique for assessing the risk of bias (RoB) in individual studies that were included in the review?** *		
**RCTs**		
For Partial Yes, must have assessed RoB from	For Yes, must also have assessed RoB from:	▯ Yes
		▯ Partial Yes
▯ unconcealed allocation, *and*	▯ allocation sequence that was not truly random, *and*	▯ No
▯ lack of blinding of patients and assessors when assessing outcomes (unnecessary for objective outcomes such as all‐cause mortality)	▯ selection of the reported result from among multiple measurements or analyses of a specified outcome	▯ Includes only NRSI
		▯ Not applicable (no study included) #
**NRSI**		
For Partial Yes, must have assessed RoB:	For Yes, must also have assessed RoB:	▯ Yes
▯ from confounding, *and*	▯ methods used to ascertain exposures and outcomes, *and*	▯ Partial Yes
		▯ No
▯ from selection bias	▯ selection of the reported result from among multiple measurements or analyses of a specified outcome	▯ Includes only RCTs
		▯ Not applicable (no study included) #
**10. Did the review authors report on the sources of funding for the studies included in the review?**		
For Yes		▯ Yes
▯ Must have reported on the sources of funding for individual studies included in the review. Note: Reporting that the reviewers looked for this information but it was not reported by study authors also qualifies		▯ No
**11. If meta‐analysis was performed did the review authors use appropriate methods for statistical combination of results?** *		
**RCTs**		▯ Yes
For Yes:		▯ No
▯ The authors justified combining the data in a meta‐analysis		▯ No meta‐analysis conducted
▯ AND they used an appropriate weighted technique to combine study results and adjusted for heterogeneity if present.		
▯ AND investigated the causes of any heterogeneity		▯ Not applicable (no study included) #
**NRSI**		
For Yes:		
▯ The authors justified combining the data in a meta‐analysis		
▯ AND they used an appropriate weighted technique to combine study results, adjusting for heterogeneity if present		▯ Yes
▯ AND they statistically combined effect estimates from NRSI that were adjusted for confounding, rather than combining raw data, or justified combining raw data when adjusted effect estimates were not available		▯ No
▯ AND they reported separate summary estimates for RCTs and NRSI separately when both were included in the review		▯ No meta‐analysis
		▯ Not applicable (no study included) #
**12. If meta‐analysis was performed, did the review authors assess the potential impact of RoB in individual studies on the results of the meta‐analysis or other evidence synthesis?**		
If a systematic review and meta‐analysis planned to do the analysis but ended up not doing because of some technical issue (e.g. no enough included studies), we chose Yes.		
For Yes:		▯ Yes
▯ included only low risk of bias RCTs		▯ No
▯ OR, if the pooled estimate was based on RCTs and/or NRSI at variable RoB, the authors performed analyses to investigate possible impact of RoB on summary estimates of effect.		▯ No meta‐analysis
		▯ Not applicable (no study included) #
**13. Did the review authors account for RoB in individual studies when interpreting/discussing the results of the review?** *		
For Yes:		▯ Yes
▯ included only low risk of bias RCTs		▯ No
▯ OR, if RCTs with moderate or high RoB, or NRSI were included the review provided a discussion of the likely impact of RoB on the results		▯ Not applicable (no study included) #
**14. Did the review authors provide a satisfactory explanation for, and discussion of, any heterogeneity observed in the results of the review?**		
For Yes:		▯ Yes
▯ There was no significant heterogeneity in the results		▯ No
▯ OR if heterogeneity was present the authors performed an investigation of sources of any heterogeneity in the results and discussed the impact of this on the results of the review		▯ Not applicable (no study included) #
**15. If they performed quantitative synthesis, did the review authors carry out an adequate investigation of publication bias and discuss its likely impact on the results of the review?** *		
For Yes:		▯ Yes
		▯ No
▯ performed graphical or statistical tests for publication bias and discussed the likelihood and magnitude of impact of publication bias		▯ No meta‐analysis conducted
		▯ Not applicable (no study included) #
**16. Did the review authors report any potential sources of conflict of interest, including any funding they received for conducting the review?**		
For Yes:		▯ Yes
▯ The authors reported no competing interests OR		
▯ The authors described their funding sources and how they managed potential conflicts of interest		▯ No

* Critical items.

# We added an option as “Not applicable” for these items when no study was included in a review.
